# 18β-Glycyrrhetinic Acid Suppresses Cell Proliferation through Inhibiting Thromboxane Synthase in Non-Small Cell Lung Cancer

**DOI:** 10.1371/journal.pone.0093690

**Published:** 2014-04-02

**Authors:** Run-Yue Huang, Yong-Liang Chu, Qing-Chun Huang, Xiu-Min Chen, Ze-Bo Jiang, Xian Zhang, Xing Zeng

**Affiliations:** 1 Department of Rheumatology, The Second Affiliated Hospital, Guangzhou University of Chinese Medicine (Guangdong Provincial Hospital of Chinese Medicine), Guangzhou, Guangdong, China; 2 Central Laboratory, The Second Affiliated Hospital, Guangzhou University of Chinese Medicine (Guangdong Provincial Hospital of Chinese Medicine), Guangzhou, Guangdong, China; Southern Illinois University School of Medicine, United States of America

## Abstract

18β-glycyrrhetinic acid (18β-GA) is a bioactive component of licorice. The anti-cancer activity of 18β-GA has been studied in many cancer types, whereas its effects in lung cancer remain largely unknown. We first showed that 18β-GA effectively suppressed cell proliferation and inhibited expression as well as activity of thromboxane synthase (TxAS) in non-small cell lung cancer (NSCLC) cells A549 and NCI-H460. In addition, the administration of 18β-GA did not have any additional inhibitory effect on the decrease of cell proliferation induced by transfection with TxAS small interference RNA (siRNA). Moreover, 18β-GA failed to inhibit cell proliferation in the immortalized human bronchial epithelial cells 16HBE-T and another NSCLC cell line NCI-H23, both of which expressed minimal level of TxAS as compared to A549 and NCI-H460. However, 18β-GA abolished the enhancement of cell proliferation induced by transfection of NCI-H23 with pCMV6-TxAS plasmid. Further study found that the activation of both extracellular signal-regulated kinase (ERK)1/2 and cyclic adenosine monophosphate response element binding protein (CREB) induced by TxAS cDNA transfection could be totally blocked by 18β-GA. Altogether, we have delineated that, through inhibiting TxAS and its initiated ERK/CREB signaling, 18β-GA suppresses NSCLC cell proliferation. Our study has highlighted the significance of 18β-GA with respect to prevention and treatment of NSCLC.

## Introduction

The herbs used in traditional medicines provide a rich reservoir for extracting biologically active compounds. For instance, licorice has been frequently used in Oriental medicine for thousands years, and its major bioactive component glycyrrhizic acid possesses diverse biological, pharmacological, and medicinal activities, which are similar to those of retinoids and steroids [1]. Glycyrrhizic acid is readily hydrolyzed to 18β-GA in human body, thereby exerting its anti-inflammatory, anti-virus and even anti-cancer effects [2]-[4]. Although chemopreventive potential of 18β-GA has been documented in many types of cancer cells, such as human epithelial ovarian carcinoma, breast cancer and glioblastoma [5]–[7], little is known about the effects of 18β-GA in lung tumor growth.

Lung cancer is the uncontrolled growth of abnormal cells in tissues of the lung. More than 1.5 million deaths worldwide are from Lung cancer, which exceed those from any other malignancies [6]. Among the many subtypes, NSCLC accounts for the 80% of all lung cancer cases [8]. Till lately there were no satisfactory therapeutic strategies for the management of NSCLC. TxAS has been observed to be over-expressed in NSCLC specimens, as compared to normal lung tissues [9]–[11]. In cancer tissues, TxAS is located downstream of cyclooxygenases (COX)-2, and it can synthesize thromboxane (Tx)-A2 from prostaglandin (PG)-H2, which is converted by COXs from arachidonic acid (AA) [12]. The biological half-life of TxA2 is about 30 s in in-vitro models, so TxA2 is rapidly and non-enzymatically degraded into an inactive form of TxB2 in aqueous solution [12], [13]. Thus, TxA2 acts as a paracrine/autocrine hormone with potent effects of platelet aggregation, vasoconstriction, tumor cell proliferation and invasion [12], [14]. The effects of TxA2 are mediated through interaction with its specific receptor, thromboxane receptor (TP), which is a member of the G-protein-coupled cell surface receptor family [12], [13]. During the past 5 years, TxAS and its related TP have been extensively studied in cancer research. The positive role of TxAS and TP in tumor pathology is therefore established, and their inhibitors/antagonists have been suggested to be the promising anti-cancer agents [10], [15]. Recently, it was reported that both TxAS and its upstream COX-2 are controlled by TP, and TxAS is able to promote lung tumor growth through this auto-regulatory feed-back loop [16]. Intriguingly, in human endothelial cells, the effects of TP agonist can be mimicked by 18β-GA with a similar time course and efficacy [17], suggesting an association between 18β-GA and molecular TxAS.

Thus, we have investigated the possible an-cancer effect of 18β-GA in NSCLC cells. We report here that 18β-GA could suppress cell proliferation and induce apoptosis in NSCLC cells through, at least in part, inhibiting TxAS expression and activity. Such effects might be expected to be of considerable clinical relevance.

## Materials and Methods

### Cell Culture and chemicals

The human lung adenocarcinoma cell lines NCI-H23, NCI-H460 and A549 as well as an immortalized human bronchial epithelial cell line 16HBE-T were purchased from the American Type Culture Collection (Rockville, MD). Both NCI-H23 and 16HBE-T cells were cultured in Dulbecco's modified Eagle's medium (DMEM), and NCI-H460 and A549 cells were cultured in RPMI 1640 medium. All cells were maintained in culture medium supplemented with 10% fetal bovine serum (FBS) in an atmosphere containing 5% CO2 at 37°C.

18β-GA and arachidonic acid were bought from Sigma-Aldrich, and cisplatin was supplied by ALADDIN Chemical Co., Ltd. (Shanghai, China).

### Cell proliferation assay

Cells were seeded at 5,000 cells per well in 96-well plates and incubated overnight. After proper treatment, the number of viable cells was quantified at the indicated time points, using MTS assay, performed in quadruplicate, according to the instruction of manufacturer (Promega, Madison, WI). Absorbance was determined using a micro plate reader at wavelength of 492 nm.

### Flow cytometric analysis

Cells were seeded at 1×10^5^ cells/10 ml in 6-well plates and incubated overnight. Live cells were collected and washed twice by ice-cold PBS. cells were stained with Annexin V fluorescein dye and propidium iodide (PI) at room temperature in dark for 15 min. cells were subsequently resuspended in 400 μl Annexin-binding buffer (Beckman Coulter, Inc., Brea, CA). The stained cells were kept on ice and analyzed by Beckman Flow Cytometers.

### Enzyme immunoassay (EIA) assay

TxAS activity was monitored by assaying the level of thromboxane B2 (TxB2), a stable product of the non-enzymatic hydration of TxA2 [12]. A549 and NCI-H460 cells were seeded at the same density of 1×10^5^ cells/10 ml of medium in a 6-well culture plate. The culture supernatant was collected and centrifuged followed the proper treatment. TxB2 was detected by peroxidase-labeled TxB2 conjugates using an enzyme immunoassay kit according to manufacturer's instruction (Cayman Chemical, Ann Arbor, MI).

### Transient transfections

Cells were seeded at the same density of 1×10^5^ cells/10 ml of medium in a 6-well culture plate. 10 nM TxAS siRNA and non-target siRNA (control) were transfected into A549 and NCI-H460, while 2 μg vacant pCMV6 (control) or pCMV6-TxAS plasmid was transfected into NCI-H23, with the aid of Lipofectamine 2000 reagents (Invitrogen, Carlsbad, CA, USA), according to the manufacturer's instruction (OriGene technologies, Rockville, MD). The extent of the specific gene knockdown or over-expression was detected by real-time PCR experiment.

The sequence of TxAS siRNA is rGrUrArArCrUrUrUrArCrCrArArCrArGrArArUrGrGrCrGTC. The conditions for siRNA transfections are as followings: cells were grown at density of 70% with the standard medium. After removal of the medium, the cells were incubated with DMEM (without antibiotics) containing premixed siRNA (10 nM) and 7.5 μl of Lipofectamine 2000 reagent, and were further incubated for 72 h.

### Reverse transcriptase (RT)-PCR and real-time quantitative PCR

RT-PCR and real-time quantitative PCR were performed as previously described [16]. The following gene-specific primer sequences were used: 5′-AATAAGAACCGAGACGAACT-3′ (sense) and 5′-GGCTTGCACCCAGTAGAG-3′ (antisense) for human TxAS; 5′-GGAAATCGTGCGTGACATT-3′ (sense) and 5′-CAGGCAGCTCGTAGCTCTT-3′ (antisense) for human β-actin. Real-time PCR was performed using the CFX96 Touch Deep Well™ Real-Time PCR Detection System (Bio-Rad Laboratories, Inc. Berkeley, CA). The fold change of control in the expression of TxAS mRNA was calculated by the 2^−ΔΔCT^ method.

### Western Blot Analysis

Total protein (20μg) was resolved by 7% SDS–polyacrylamide gel electrophoresis and subjected to western blot analysis using the most advanced chemiluminescent detection system (Bio-Rad Laboratories). Western blots were probed with a mouse monoclonal antibody against TxAS (OriGene technologies), a goat polyclonal antibody against β-actin (Santa Cruz Biotechnology), and the rabbit polyclonal antibodies against GAPDH, PARP, total and phosphorylated ERK1/2 as well as total and phosphorylated CREB (Cell Signaling Technology, Beverly, MA). To ensure equal protein loading, membranes were stripped and subsequently probed with anti-GAPDH or anti-β-actin antibodies.

### Statistical Analyses

Data are depicted as means±SD from at least three independent experiments. Student's t test was used for the comparisons between two groups, while One-way ANOVA followed by Dunnett's test was adopted to compare the differences among more than two groups. Statistical analyses were performed by SPSS 11.6 statistical software (SPSS, Chicago, IL). A two-tailed P value of <0.05 was considered to be statistically significant for all experiments.

## Results

### 18β-GA suppressed cell proliferation via induction of apoptosis

**Figure 1:**
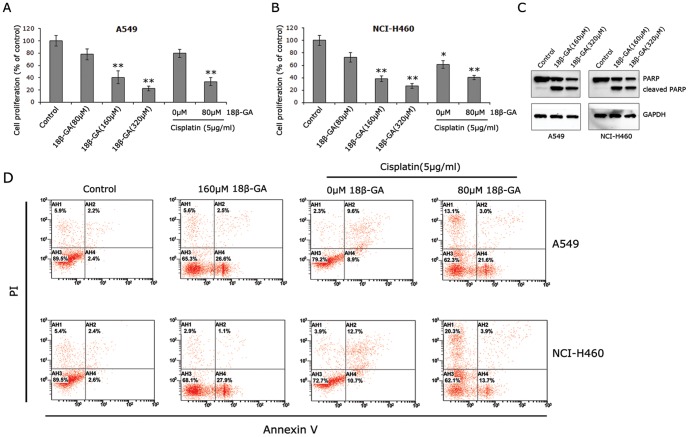
Effects of 18β-GA on cell proliferation and apoptosis. **A** and **B**, MTS experiments showed that 18β-GA inhibited cell proliferation of A549 and NCI-H460 cells in a dose-dependent manner. Moreover, 18β-GA increased cytotoxicity of chemotherapeutic agent cisplatin. Data are expressed as mean±SD of three independent experiments done in triplicate. *p<0.05, **p<0.01. **C**, Western blot analysis demonstrated the increased cleaved PARP (85 kDa) with the decreased full length PARP (116 kDa) in A549 and NCI-H460 cells treated with 18β-GA. GAPDH (37 kDa) served as the loading control. **D**, Flow cytometric analysis of cell apoptosis. Percentage of cells in early or late apoptosis is provided in the lower right and upper right quadrants, respectively. Figures are the representative result selected from three independent experiments.

Both A549 and NCI-H460 are NSCLC cell lines. The former belongs to adenocarcinoma, and the latter is a large cell lung cancer cell line. Assessment of the number of viable cells by MTS assay demonstrated that 24 h treatment of 18β-GA suppressed cell proliferation in both cell lines in a dose-dependent manner (Figure 1A). Previous in-vitro study showed that the IC_50_ value of 18β-GA for inhibiting lung cancer cell growth was 145.3 μM [18]. As shown in Figure 1A and 1B, 18β-GA at 160 μM significantly decreased the percentage of viable cells to around 40.5±10.5% in A549 and 38.3±4.6% in NCI-H460 (p<0.01 respectively). When the cells were treated with 320 μM 18β-GA, a greater inhibitory effects on cell proliferation was shown, as the percentage of viable cells was below 30% compared with untreated controls (p<0.001). Therefore, it appeared that 160 μM 18β-GA was an optimal concentration to achieve a significant suppression of cell proliferation in NSCLC cells.

Cisplatin is part of the standard of care for patients with lung cancer. To determine the possibility that 18β-GA could have an additive effect to cisplatin on tumor cell proliferation, both cell lines tested were grown in the presence of 5 μg/ml cisplatin alone or 5 μg/ml cisplatin together with 80 μM 18β-GA. When treated with cisplatin alone, the percentage of viable cells was only decreased by 20.5±6.8% in A549 and 38.7±6.0% in NCI-H460 compared to control (p = 0.076 and 0.041 respectively, Figure 1A and 1B). However, when combined with 80 μM 18β-GA, they had a synergistic effect in both cell lines. In both cell lines, the suppression rate after combined treatment was below 40% of controls (p<0.01), similar to the efficacy of 160 μM 18β-GA alone.

To confirm the data observed by MTS assays and to determine whether suppression of cell proliferation was in part due to an increase in apoptosis, cleaved PARP was measured by Western blotting using a rabbit polyclonal PARP antibody detecting both full-length and cleaved forms. As illustrated in figure 1C, treatment with 18β-GA at 160 μM and 320 μM decreased the levels of full-length PARP and increased the levels of cleaved-PARP. This result was further supported by flow cytometric analysis which was used to measure annexin-V labeling to phosphatidylserine, a membrane phospholipid exposed at the surface of apoptotic cells [19]. Following 24 h treatment with 160 μM 18β-GA, cells were stained with annexin-V-fluorescein isothiocyanate and PI. As illustrated in figure 1D, the fraction of cells in early apoptosis, indicated by PI-positive cells, was significantly higher in 18β-GA-treated groups (about 3.5-fold for A549, and about 3.9-fold for NCI-H460, respectively; P<0.001). In addition, more apoptotic cells were found in treatment of cisplatin combined with 18β-GA than treatment of cisplatin alone, further supporting that 18β-GA has an additive effect to cisplatin.

### 18β-GA inhibited TxAS expression and activity

**Figure 2:**
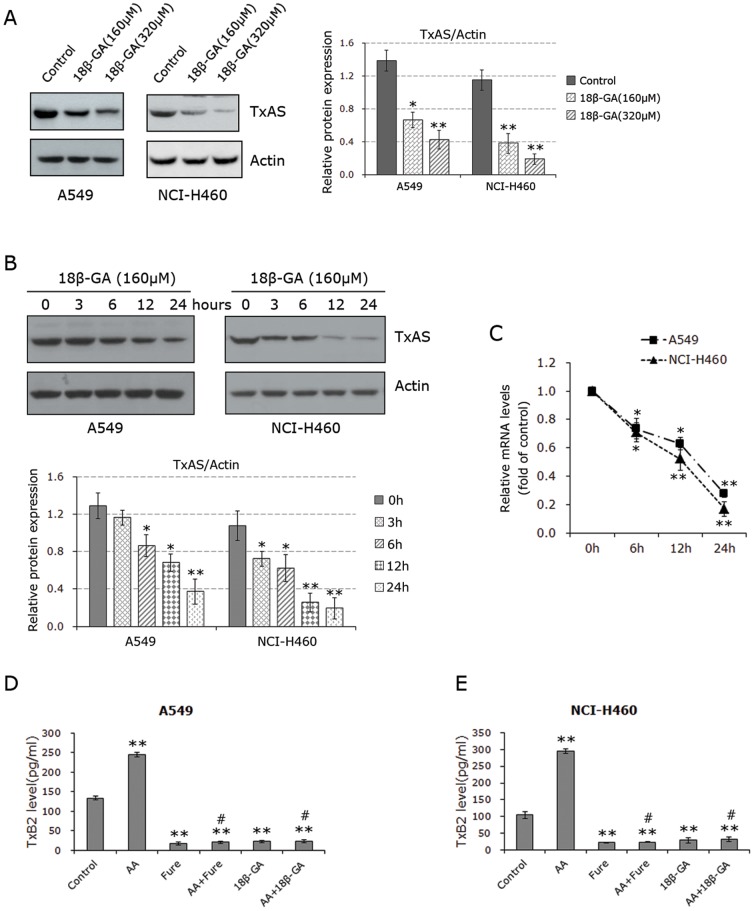
Effects of 18β-GA on TxAS expression and activity. **A** and **B,** Western blot analysis found that 18β-GA inhibited protein expression of TxAS (60 kDa) in a dose- and time-dependent manner. Actin (45 kDa) served as the loading control. Figure shown is representative of three independent experiments, and densitometry for blots was shown in the right panel of fig.A and the lower panel of fig.B. *P< 0.05 and **P< 0.01 as compared to control. **C**, real-time PCR revealed that the expression of TxAS mRNA could be reduced by 18β-GA in a time-dependent manner. Dare are expressed as the fold of control, which was calculated by 2^−ΔΔCT^ method based on the data observed from three independent experiments done in triplicate. *P< 0.05 and **P< 0.01. **D** and **E**, TxB2 EIA assays demonstrated the effects of 18β-GA on TxAS activity in both A549 and NCI-H460 cells in the absence or presence of 0.4μM AA. Data are expressed as mean±SD of three independent experiments done in triplicate. **p<0.01 when compared to control, # p<0.01 as compared with AA treatment.

Previous studies implicated a potential role of TxAS in the pathogenesis of several different types of cancer, including NSCLC [10], [16]. We therefore examined if 18β-GA could affect TxAS expression and activity. Western blot analysis showed that 18β-GA decreased TxAS protein level in a time- and dose-dependent manner (Figure 2A and 2B). Consistent with apoptotic effect of 18β-GA shown in figure 1, 12 h treatment of 18β-GA at 160 μM and 320 μM dramatically decreased the levels TxAS. Moreover, 18β-GA (160 μM) inhibited the level of TxAS as early as 3 h in NCI-H460 and 6 h in A549 after the treatment. mRNA was also extracted from cells treated with 160 μM 18β-GA for 6 h, 12 h and 24 h and subsequently subjected to real-time PCR experiments. Figure 2C showed that TxAS mRNA level was significantly decreased by 18β-GA in a time-dependent manner, which is in line with the data observed by Western blot analysis.

Moreover, the effect of 18β-GA on TxAS activity was measured subsequently. As aforementioned, TxA2 is chemically unstable in-vitro, TxAS activity is therefore monitored by assaying the level of TxB2, a stable product of the non-enzymatic hydration of TxA2 [16]. Both A549 and NCI-H460 cells were treated with 160 μM 18β-GA for 24 h, and the culture medium was collected to perform TxB2 EIA assay. In these experiments, the cells treated with 0.4 μM AA which is the precursor of TxA2 served as the positive controls, and the cells treated with specific TxAS inhibitor furegrelate (1 mM) served as the additional controls. The selected doses of these chemicals are comparable to the concentrations used in in-vitro experiments reported in other studies [16], [20]. As shown in figure 2D and 2E, in both cell lines, the biosynthesis of TxA2 was significantly suppressed by 18β-GA in the absence or presence of AA. 0.4 μM AA induced TxB_2_ production by 1.8-fold in A549 cells (p<0.01) and 2.8-fold in NCI-H460 cells (p<0.01) as compared to control. However, the addition of 160 μM 18β-GA significantly attenuated AA-induced TxB_2_ production by nearly 91% in A549 and 89% in NCI-H460 (p<0.001 respectively). Importantly, 18β-GA at 160 μM is the equivalent in efficacy to 1 mM furegrelate. These results strongly suggest that 18β-GA is able to abrogate TxAS activity in NSCLC cells.

Together, the data of that 18β-GA could significantly inhibit both expression and activity of TxAS implies that 18β-GA may suppress cell proliferation through inhibiting TxAS action.

### 18β-GA had no additional effects on cell proliferation when TxAS was knocked down

**Figure 3:**
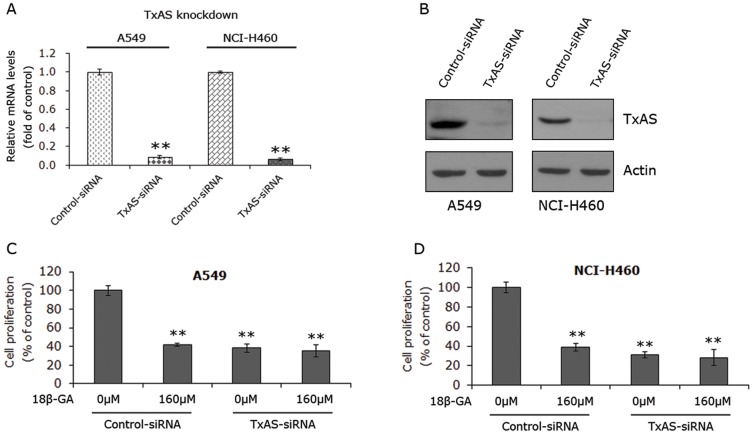
Effects of 18β-GA on cell proliferation in cells transfected with TxAS-siRNA. **A** and **B**, the suppressing efficacy of siRNA on TxAS expression was revealed by real-time PCR and Western blot analysis. Real-time PCR data were observed from three independent experiments done in triplicate and calculated by 2^−ΔΔCT^ method. Data are presented as the fold of control, **P<0.01. **C** and **D**, MTS assays showed that TxAS-siRNA transfection inhibited cell proliferation in A549 and NCI-H460, and the additional administration of 18β-GA had no additive effects. Data are presented as percentages of the control and expressed as mean±SD of three independent experiments done in triplicate. **p<0.01.

To verify the possibility that 18β-GA may suppress cell proliferation through inhibiting TxAS, we knocked down TxAS expression in A549 and NCI-H460 cells using siRNA transfection and 160 μM 18β-GA was subsequently added for 24 h. Non-target siRNA was also transfected into both cell lines to serve as the control. Real-time PCR indicated that the expression of TxAS in both cell lines was significantly suppressed by 24 h transfection of TxAS-siRNA, with nearly 90% of suppression efficacy (figure 3A). The transfection efficacy was further confirmed by Western blot analysis (figure 3B). After the addition of 160 μM 18β-GA for another 24 h, MTS assays were performed and the results showed that the proliferation rates of A549 cells treated with 18β-GA, TxAS-siRNA, and the combination of both were 41.7±1.7%, 38.3±4.4% and 35.4±6.4% of controls respectively (figure 3C). In NCI-H460 cells, they were 38.9±3.7%, 31.1±3.4% and 28.4±8.0% of controls, respectively (figure 3D). It appears that compared with TxAS-siRNA transfection, the additional administration of 18β-GA did not have any significant additional effects on cell proliferation.

Of note, the result that siRNA of TxAS significantly reduced cell growth, which is in line with the data observed in our previous studies showing that specific TxAS inhibitor furegrelate can dramatically inhibit cell growth in lung cancer cells [11], [16]. In support of our data, Cathcart MC et al showed that another TxAS inhibitor Ozagrel inhibited cell proliferation via induction of apoptosis in NSCLC cells [10], and Moussa O et al showed that TxAS shRNA suppressed bladder cancer cell growth [19]. Moreover, Nie D et al observed that prostate tumor cell motility was attenuated by inhibitors of TxAS [21].

### Suppression of cell proliferation by 18β-GA was associated with TxAS status

**Figure 4:**
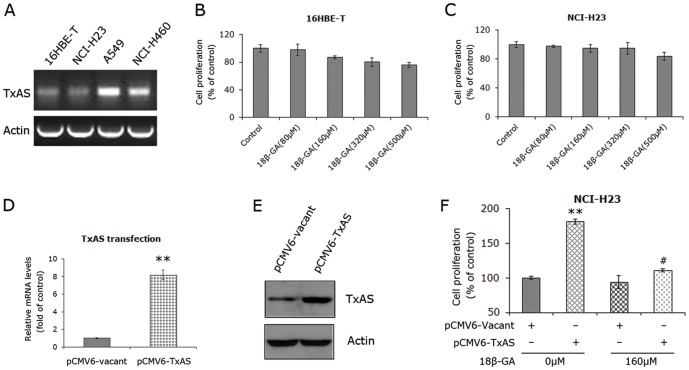
Examination of role of TxAS in 18β-GA effects. **A**, the minimal level of TxAS in 16HBE-T and NCI-H23 and the over-expression of TxAS in A549 and NCI-H460 were revealed by RT-PCR. Figure shown is representative of three independent experiments. **B** and **C**, MTS demonstrated that while there was a slight inhibitory trend, 18β-GA failed to control cell proliferation in 16HBE-T and NCI-H23. Data are presented as percentages of the control and expressed as mean±SD of three independent experiments done in triplicate. **D** and **E**, TxAS expression in NCI-H23 was over-expressed by transfection with pCMV6-TxAS plasmid. Real-time PCR data were observed from three independent experiments done in triplicate and calculated by 2^−ΔΔCT^ method. Data are presented as the fold of control, **P<0.01. **F**, MTS assays showed that the promotion of cell proliferation by transfection with pCMV6-TxAS was abrogated by 18β-GA. Data are presented as percentages of the control and expressed as mean±SD of three independent experiments done in triplicate. **p<0.01 when compared to control, #p<0.01 as compared with pCMV6-TxAS transfection.

The results observed above supported that the inhibitory effects of 18β-GA may be due to the suppression of TxAS in NSCLC cells. To further verify this possibility, we next screened a series of lung cell lines for the expression of TxAS. RT-PCR experiments showed that, compared to A549 and NCI-H460, an immortalized human bronchial epithelial cell line 16HBE-T and another NSCLC cell line NCI-H23 expressed minimal level of TxAS (figure 4A). These two cell lines were treated with increasing concentrations of 18β-GA for 24 h, and MTS assay was performed to detect the cell proliferation. As shown in figure 4B and 4C, 18β-GA failed to abolish cell proliferation in both 16HBE-T and NCI-H23, albeit with a slight trend of suppression. The suppression rate at dose of 500 μM was not more than 20% of control in both cell lines.

Because NCI-H23, belonging to adenocarcinoma, is a NSCLC cell line with the minimal level of TxAS, we over-expressed TxAS in this cell line by transfection with pCMV6-TxAS plasmid. Vacant plasmid was also transfected to serve as the control. Figure 4D illustrated that TxAS was over-expressed about 8.2-fold than control after 24 h transfection with TxAS cDNA. Western blot analysis was also performed to confirm the transfection efficacy (figure 4E). Cells were subsequently treated with 160 μM 18β-GA for another 24 h. MTS experiments demonstrated that transfection with TxAS cDNA markedly promoted cell proliferation by 181.4±3.6% as compared to the control (p<0.001), the effect was abolished by addition of 18β-GA. The cell proliferative rate was significantly decreased by 18β-GA to 111.2±2.2% of control, almost even with the control level (figure 4F). These findings support that the effect of 18β-GA in NSCLC is associated with the change of TxAS expression.

### ERK/CREB signaling was involved in 18β-GA effects

**Figure 5:**
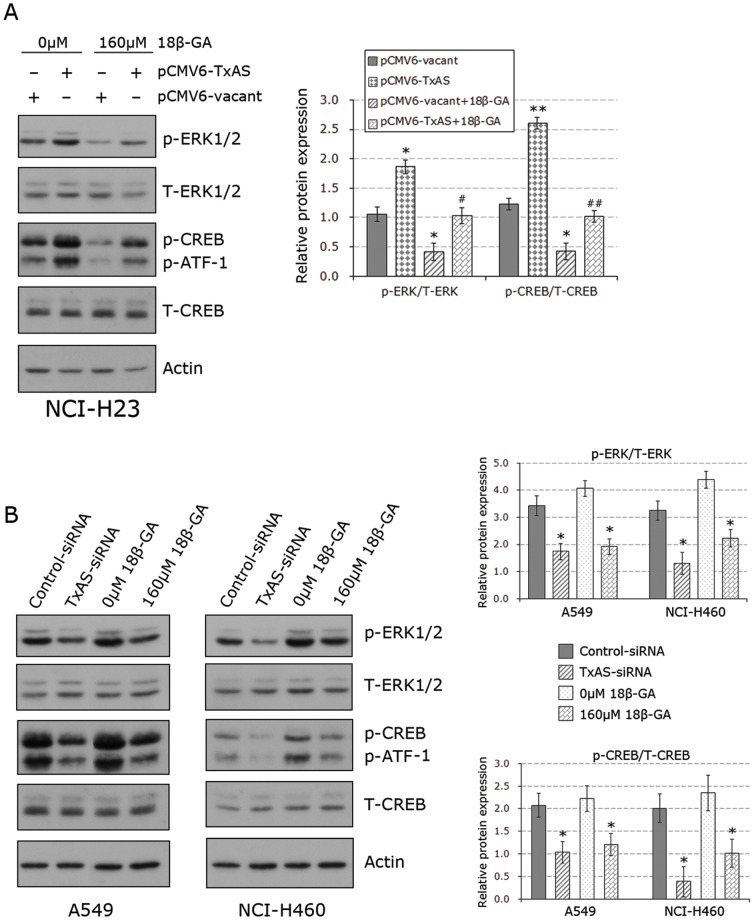
Effects 18β-GA on ERK and CREB activation. **A**, Western blotting experiments demonstrated that the activation of ERK1/2 (42/44 kDa) and CREB (43 kDa) induced by pCMV6-TxAS transfection could be abolished by 18β-GA in NCI-H23 cells. Figure shown is representative of three independent experiments, and densitometry for blots was shown in the right panel. *p<0.05 **p<0.01 as compared to control; # p<0.05 and ## p<0.01 when compared with 18β-GA treatment in cells transfected with vacant plasmid. **B**, the suppression of ERK1/2 and CREB activation by TxAS-siRNA and 18β-GA in A549 and NCI-H460 was revealed by Western blot analysis. In these experiments, total ERK1/2 (42/44 kDa), total CREB (43 kDa) and Actin (45 kDa) served as loading controls. A monoclonal antibody that can detect endogenous levels of p-CREB and the phosphorylated form of the CREB-related protein activating transcription factor-1 (p-ATF-1) was used in the current study. Figure shown is representative of three independent experiments, and densitometry for blots was shown in the right panel. *p<0.05 as compared to control.

In our previous studies, we demonstrated that the activation of both ERK and CREB can be inhibited by TxAS inhibitors or TP antagonists, and activation of CREB is suppressed by specific ERK inhibitor [11], [16]. TxAS/ERK/CREB pathway is also addressed by other studies done in lung cancer model [22], [23]. Therefore, the final series of Western blotting experiments were done to investigate if ERK/CREB pathway was involved in the tumor-suppression effects of 18β-GA. As shown in figure 5A, the cells transfected with pCMV6-TxAS presented the higher levels of phosphorylated ERK1/2 (p-ERK1/2) and phosphorylated CREB (p-CREB), the effects were dramatically attenuated by treatment of 160 μM 18β-GA. The result is in line with the data observed by MTS assays (figure 4F). In addition, we determined effects of 18β-GA on ERK/CREB activation in A549 and NCI-H460, both of which present high levels of TxAS as seen in figure 4A. Figure 5B showed that the down-regulation of TxAS by siRNA blocked ERK1/2 phosphorylation in A549 and H460 cells. Consistently, 160 μM 18β-GA effectively suppressed the high levels of p-ERK and p-CREB in these two cell lines. No alteration was detectable regarding the expression of total ERK or total CREB in all cell lines tested.

## Discussion

As one of herbs frequently used in Oriental medicine with low toxicity, licorice is a US Food and Drug Administration (FDA) approved food supplement used in many products. As stated above, glycyrrhizic acid is the major bioactive component and it is readily hydrolyzed to be 18β-GA in human body to exert diverse effects, including anti-cancer activity [1]–[4]. This study demonstrated the tumor-suppressing effect of 18β-GA in NSCLC cells, and TxAS was found to be implicated in this effect.

We initially showed that 18β-GA dose-dependently decreased cell proliferation in NSCLC cells A549 and NCI-H460. The ID50 of 18β-GA in HepG2 cells was 80 μM [24], while in our model the dose of 160 μM decreased cell proliferation rate under 50% of control, which is in line with a previous report showing that the IC_50_ value of 18β-GA was 145.3 μM in a highly potentially metastatic lung cancer cell line PGCL3 [18]. 80 μM 18β-GA in combination with cisplatin yielded a significant tumor-suppressing effect, in spite of 18β-GA alone at 80 μM had no effective efficacy to kill cancer cells. This result suggests the synergistic effect of 18β-GA on chemotherapeutic agent. A549 showed higher resistance to cisplatin compared with NCI-H460 cells, which is in accordance with other studies demonstrating that A549 is more resistant to cisplatin than some other cell lines [25], [26]. Western blot analysis further presented the increased cleaved-PARP with the decreased full-length PARP in cells treated with 18β-GA at the doses of 160 μM and 320 μM. Thus, the change in total viable cell number at 24 h after treatment with 18β-GA noted in MTS experiments can be attributed to increased apoptosis, which is confirmed by the data observed by flow cytometric analyses. Collectively, it appears that 18β-GA is a promising anti-cancer agent for application in NSCLC prevention and treatment.

We proceeded to examine the molecular mechanisms whereby 18β-GA exerts tumor-suppressing effect in NSCLC cells. By virtue of catalysing the formation of TxA2 which functions through its receptor TP, TxAS has been shown to have a potential role in the cancer phenotype [10], [11], [14]–[16]. Importantly, TxAS and its product TxA2 were found to be overexpressed in lung cancer tissues, as compared to normal lung tissues [9]-[11], [27]. A previous report showing that 18β-GA is the equivalent in efficacy to a TP agonist in human endothelial cells [17] led us to ask if TxAS was implicated in 18β-GA effects in NSCLC. This hypothesis was also enlightened by a fact that glycyrrhizic acid, the precursor of 18β-GA, acts in part through inhibiting COX-2 [28], which provides PGH2 for TxAS to catalyse TxA2 formation [12], [29]. Coupled with the results that 24 h treatment of 18β-GA dose-dependently suppressed cell proliferation and induced apoptosis, the decrease of TxAS by 12 h treatment of 18β-GA suggests that the 18β-GA -induced inhibition of TxAS is an upstream event of growth inhibition and apoptosis in NSCLC, which is further supported by the following time course experiments. In addition, the mRNA levels of TxAS could be reduced by 18β-GA in a time-dependent manner, implying that 18β-GA inhibited TxAS expression at transcriptional level. Furthermore, TxAS activity, reflected by TxB2 level secreted into culture medium, was dramatically inhibited by 18β-GA. These results suggest that TxAS could be a crucial molecular basis of 18β-GA effect in NSCLC cells. By transfection with TxAS-siRNA, cell proliferative ability of both A549 and NCI-H460 was effectively inhibited, further supporting the positive role of TxAS in lung cancer [10], [11], [16]. Importantly, the additional administration of 18β-GA did not have the additive effects on TxAS-siRNA transfection. To confirm these results, we screened a series of lung cell lines to determine the expression of TxAS. NSCLC is any type of epithelial lung cancer other than small cell lung carcinoma (SCLC), so we used 16HBE-T which is an immortalized human bronchial epithelial cell line to serve as control for NSCLC cell lines. In accordance with other studies [9]–[11], TxAS level was found to be minimal in 16HBE-T, while it was over-expressed in NSCLC cells A549 and NCI-H460. Another NSCLC cell line NCI-H23 also expressed minimal level of TxAS, making it ideal model for transfection of TxAS cDNA. As expected, 18β-GA failed to effectively suppress cell proliferation of 16HBE-T and NCI-H23. It appeared that the different effects of 18β-GA in all of these cell lines were due to the difference of TxAS expression. Therefore, all of these results suggest that 18β-GA suppressed cell proliferation through, at least in part, inhibiting TxAS expression and activity. The conclusion was further confirmed by the data showing that the enhancement of cell proliferation by transfection with TxAS cDNA in NCI-H23 cells could be totally abrogated by the addition of 18β-GA.

Multiple intracellular signalings mediate the cancer phenotype. CREB is a key transcriptional factor, and it is thought to have a central role in tumor pathogenesis [30]. We previously demonstrated that ERK was identified to be mediated by TxA2 and CREB was located downstream of ERK pathway in lung cancer cells. Moreover, CREB siRNA could totally inhibit cell proliferation in lung cancer cells [11], [16]. In addition, many reports also have clarified that CREB is located downstream of ERK and is responsible for the promoting effects of TxA2 on cell proliferation [22], [23]. At last, we proved the involvement of ERK/CREB pathway in 18β-GA effect, as shown by the data that treatment with 18β-GA effectively blocked activation of either ERK or CREB induced by TxAS cDNA transfection in NCI-H23 cells. The results were further confirmed by data observed in A549 and NCI-H460 cells.

In summary, we demonstrated, for the first time, that 18β-GA suppressed ERK/CREB pathway via inhibiting TxAS, thereby decreasing cell proliferation of NSCLC cells. It is noteworthy that, as stated above, TxAS is over-expressed in NSCLC and carries a poor prognosis [10], [11]. This is of great importance since 18β-GA significantly suppressed cell proliferation only in A549 and NCI-H460 cells which express high level of TxAS. In addition, the nephrotoxicity induced by cisplatin is thought to be mediated in part through the TxA2 pathway [19], [31], which provides further support for that the use of 18β-GA in addition to cisplatin may provide a protective effect from the adverse side-effect of cisplatin. Now there is a global attempt to look for a potential anti-cancer agent for treatment of lung cancer with the low toxicity. Our study presented here suggests that 18β-GA could be an ideal candidate. Further studies done in-vivo will be needed to verify the tumor-suppressing effect of 18β-GA.
